# Gait Improvement in Chronic Stroke Survivors by Using an Innovative Gait Training Machine: A Randomized Controlled Trial

**DOI:** 10.3390/ijerph19010224

**Published:** 2021-12-25

**Authors:** Patcharee Kooncumchoo, Phuwarin Namdaeng, Somrudee Hanmanop, Bunyong Rungroungdouyboon, Kultida Klarod, Sirirat Kiatkulanusorn, Nongnuch Luangpon

**Affiliations:** 1 Department of Physical Therapy, Faculty of Allied Health Sciences, Thammasat University, Pathumthani 12120, Thailand; patcharee.k@allied.tu.ac.th (P.K.); phuwarin.n@allied.tu.ac.th (P.N.); somrudee.h@allied.tu.ac.th (S.H.); 2 Center of Excellence in Creative Engineering Design and Development, Thammasat University, Pathumthani 12120, Thailand; rbunyong@engr.tu.ac.th; 3 Department of Mechanical Engineering, Faculty of Engineering, Thammasat University, Pathumthani 12120, Thailand; 4 Department of Physical Therapy, Faculty of Allied Health Sciences, Burapha University, Chonburi 20131, Thailand; kultida@go.buu.ac.th (K.K.); siriratk@go.buu.ac.th (S.K.)

**Keywords:** stroke, gait training, lower limb impairment, motor recovery

## Abstract

Chronic stroke leads to the impairment of lower limb function and gait performance. After in-hospital rehabilitation, most individuals lack continuous gait training because of the limited number of physical therapists. This study aimed to evaluate the effects of a newly invented gait training machine (I-Walk) on lower limb function and gait performance in chronic stroke individuals. Thirty community-dwelling chronic stroke individuals were allocated to the I-Walk machine group (*n* = 15) or the overground gait training (control) group (*n* = 15). Both groups received 30 min of upper limb and hand movement and sit-to-stand training. After that, the I-Walk group received 30 min of I-Walk training, while the control followed a 30-minute overground training program. All the individuals were trained 3 days/week for 8 weeks. The primary outcome of the motor recovery of lower limb impairment was measured using the Fugl–Meyer Assessment (FMA). The secondary outcomes for gait performance were the 6-minute walk test (6 MWT), the 10-meter walk test (10 MWT), and the Timed Up and Go (TUG). The two-way mixed-model ANOVA with the Bonferroni test was used to compare means within and between groups. The post-intervention motor and sensory subscales of the FMA significantly increased compared to the baseline in both groups. Moreover, the 6 MWT and 10 MWT values also improved in both groups. In addition, the mean difference of TUG in the I-Walk was higher than the control. The efficiency of I-Walk training was comparable to overground training and might be applied for chronic stroke gait training in the community.

## 1. Introduction

Stroke is a major cause of death and global disability [1]. Thirty percent of stroke survivors are incapable of walking independently [2]. Gait training by physical therapists is mainly available only in the poststroke acute phase for a short period [3]. Gait speed is an important indicator of community ambulation associated with walking competency. Greater gait speed suggests good functioning and better quality of life [4,5]. Ng et al. found that stroke patients with a gait impairment appeared to walk at a slower pace in the 10-meter walk test and a shorter length in the 6-minute walk test than healthy people [6]. As a result of the chronic phase of stroke (≥6 months), 50% of community-dwelling persons with hemiparesis fail to complete a 6-minute walk test (6 MWT), and they are only able to walk half the distance of their predicted values [7]. Gait training methods can be classified into conventional overground gait training, staircase gait training, and device-assisted gait training. Overground gait training is one of the most common interventions for poststroke individuals. It can be defined as a physical therapist-guided gait pattern together with related exercises with no high-technology assistive devices [8]. Meanwhile, staircase gait training is used to enhance cardiovascular endurance and is suitable for patients with good dynamic balance. On the other hand, patients with poor stability need assistive devices, such as partial weight support belts.

Previous studies have reported the benefits of overground, treadmill, staircase, and device-assisted gait training on motor and sensory lower limb function, walking endurance, and walking speed in stroke survivors [9,10]. Gama et al. reported that the effects of overground training on motor function were superior to treadmill training in chronic stroke individuals [10]. Park and colleagues showed that step climbing and staircase walking exercise improved walking speed in chronic stroke patients, reducing the Timed Up and Go (TUG) test [11]. Moreover, Donath et al. reported that the staircase walking exercise enhanced cardiovascular endurance in healthy older individuals, indicated by a reduced resting heart rate [12]. Bizovičar et al. found that a motorized gait assistive device improved motor recovery measured by the Fugl-Meyer Assessment (FMA) in acute-chronic stroke patients after three weeks [9]. Pansuksawat et al. suggested that the I-Walk machine improved gait speed, motor impairment, the lower extremity joint angle, and the gait kinematic variables in chronic stroke [13].

Neuroplasticity training in hemiparesis after a stroke consists of five principles—specificity, repetition, intensity, time, and salience [14]. Therefore, optimal training procedures should have these properties. Indeed, other tools or models of therapy may need to be considered rather than the traditional one-on-one interaction. Group or robot-assisted therapies are areas currently under investigation that support the goal of an increased number of repetitions [15]. This study aimed to examine the effects of an innovative gait training machine (I-Walk) on motor control and gait performance in community-dwelling persons with hemiparesis after a stroke.

## 2. Materials and Methods

### 2.1. Study Design

**Figure 1:**
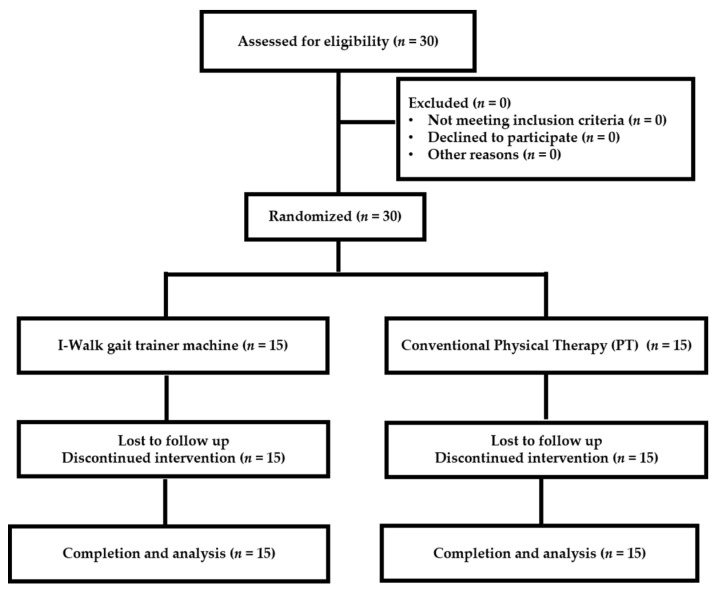
Flowchart representing the number of patients throughout the trial measures.

The study design was a randomized controlled trial. All participants were trained for 8 weeks, and FMA, 6 MWT, TUG, and 10 MWT measurements were carried out on weeks 0 and every 2 weeks by a single-blinded investigator throughout the study. An Ethical Review was approved by University Sub-Committee Board for Human Research (103/2556). Block randomization was performed, and the participants were divided into the following two groups: control group (overground training; conventional physical therapy (PT), *n* = 15) and I-Walk gait trainer machine group (*n* = 15) (Figure 1). One day before the study, the agreement to the study protocol, methods, and measurements was ensured.

### 2.2. Participants

Thirty participants who matched the criteria were recruited from two communities. Inclusion criteria were (1) a first-time stroke with more than 6 months post-stroke, (2) aged between 40 and 70 years, (3) able to walk more than 3 m with or without gait devices, and (4) able to understand or follow commands. Exclusion criteria were (1) unstable cardiovascular problems and (2) musculoskeletal disorders that altered the training program.

### 2.3. Intervention

**Figure 2:**
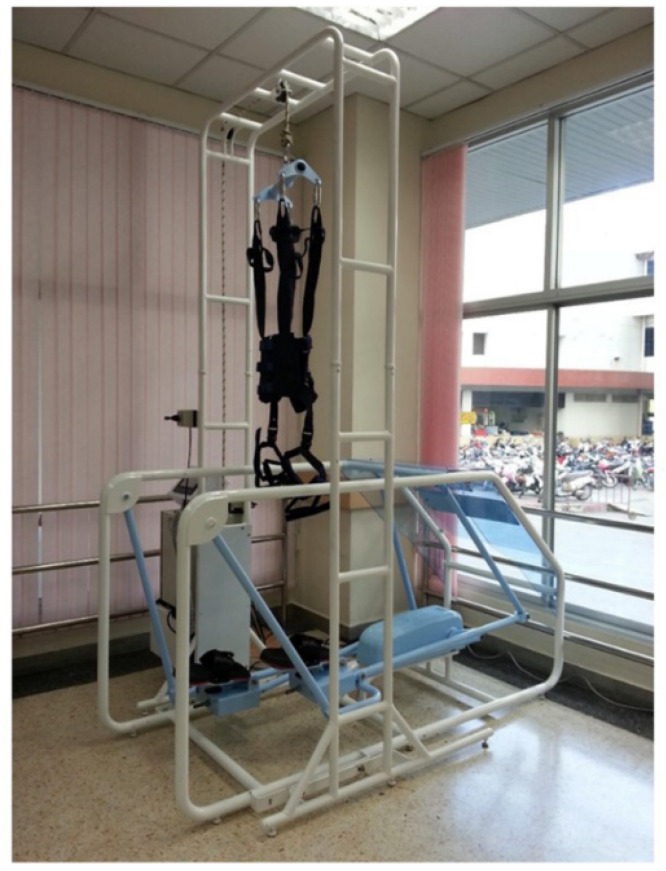
PT rehabilitation program with I-Walk machine.

The I-Walk machine (Cmed Medical, Pathumthani, Thailand) was designed as a specific tool for assisting walking and controlling walking patterns (staircase walking) with an adjustable number of repetitive walking steps (0–120 steps/min) [13]. Recoil spring plates were situated in the plantar area and were designed to counteract the ground force, assisting talocrural joint movement. The features of the closed kinematic chain allowed reciprocal hip and knee joint movement. The weight support device was adjustable based on the user’s weight-bearing ability (Figure 2). Participants in this group first received upper limb and hand movement exercises and sit-to-stand training for 30 min followed by gait training with the I-Walk gait trainer machine. All participants started gait repetition from 50 steps/min for 30 min, 3 days/week for 8 weeks by the first physical therapist. The intensity and duration could be adjusted according to the individual’s capability.

In Conventional Physical Therapy (PT), the control group first received upper limb and hand movement and sit-to-stand training for 30 min followed by overground training for 30 min, 3 days/week for 8 weeks, by a physical therapist.

### 2.4. Outcome Measures

#### 2.4.1. Primary Outcomes

Gait performances were evaluated by the 6-minute walk test (6 MWT) for walking distances and cardiovascular endurance, the 10-meter walk test (10 MWT) for gait speed, and the TUG for detecting dynamic balance during walking at the same points with the primary outcome.

#### 2.4.2. The 6-Minute Walk Test (6 MWT)

The 6 MWT has been used to define and monitor gait performance changes in walking endurance and cardiovascular function to predict community ambulation in stroke survivors. Basic individual demographics are important factors [6]. It measures the distance one can walk at a self-selected pace on a flat, hard surface in 6 min [16].

#### 2.4.3. The 10-Meter Walk Test (10 MWT)

The 10 MWT has been designed to measure walking or gait speed. It requires a 20-meter, indoor, flat, straight hallway. The first and last 5 m are used for walking acceleration and deceleration, while the time during the middle 10-meter distance is recorded. The participants are asked to walk at a self-selected speed with or without walking aids such as a walker or cane. The speed was calculated by dividing the distance by time (m/s). The test has excellent internal consistency and reliability for individuals with chronic stroke [17].

#### 2.4.4. Timed Up and Go

The TUG, developed by Podsiadlo and Richardson, evaluates functional mobility and dynamic balance while walking [18]. The test records the time it takes for a participant to complete the consecutive test of standing up from a chair, walking 3 m forward, turning around, and sitting back down on the chair to assess the individual’s dynamic balance and ambulation [17].

#### 2.4.5. Secondary Outcome

The secondary outcome of lower limb motor recovery was measured by the FMA at baseline, 2 weeks, 4 weeks, 6 weeks, and at the end of the experiment. The test has high internal consistency and reliability, excellent responsiveness, and high content validity in the population with chronic stroke [19,20,21]. Details of the outcome measurements have been explained elsewhere [13,22].

### 2.5. Statistical Analysis

Data were analyzed using SPSS version 17.0 (SPSS Inc., Chicago, IL, USA). All the data are presented as mean ± SD unless otherwise indicated. A two-way mixed-model ANOVA was used to compare all variables within and between groups. Post hoc analysis was conducted using the Bonferroni test, then the statistical approach was tested with a *t*-test. The significance level was set at 0.05. The sample size was calculated based on a previous study [23].

## 3. Results

Thirty participants completed gait performance training (15 overground training as conventional PT and 15 I-Walk training). All the baselines between both groups were not statistically different, as shown in Table 1.

### 3.1. Effects of Gait Performance Training Measured Using the Fugl-Meyer Assessment of Lower Extremity Scores in I-Walk and Conventional PT Training

Six sub-scores of the FMA of lower extremity (FMA-LE) scores of the participants who trained with I-Walk and the overground method are shown in Table 2. Compared with baseline, the total FMA-LE scores significantly increased in both groups (F(4,112) = 75.5; *p* < 0.001; effect size (ES) = 0.73), then the statistical approach with the *t*-test started to increase at 2 weeks (*p* < 0.05). Meanwhile, the total motor function in both groups significantly increased (F(4,112) = 88.8; *p* < 0.001; ES = 0.76), while it began to increase at 2 weeks for the I-Walk group (*p* < 0.05). There was a main time effect in lower limb sensation for both groups in the present study with a repeated measure ANOVA (F(4,112) = 13.9; *p* < 0.001; ES = 0.33). The conventional PT was significantly higher in lower limb sensation, which began at 2 weeks (*p* < 0.05), but the I-Walk group started to increase at 4 weeks (*p* < 0.05). Unexpectedly, the passive joint motion sub-score significantly increased only in the conventional PT group (F(4,112) = 16.9; *p* < 0.001; ES = 0.38). No significant change was observed for the joint pain sub-score.

In the comparison across groups, no difference was observed in the four lower extremity sub-scores (coordination/speed, total motor function, sensation, joint pain). Two sub-scores (lower extremity and passive joint motion) in the I-Walk group were higher than the conventional PT score at baseline (*p* < 0.05). However, conventional PT tended to gain in passive joint motion compared to I-Walk.

### 3.2. Effect of Gait Performance, Dynamic Balance, and Gait Speed in Chronic Stroke

**Figure 3:**
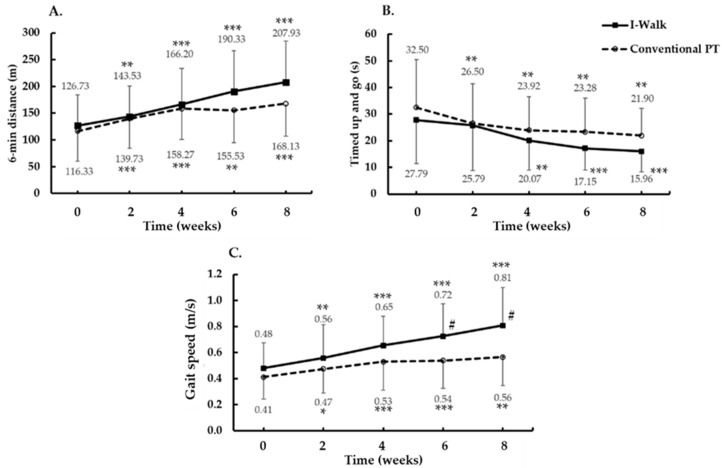
Gait performance, dynamic balance, and gait speed changes after gait trainings for 8 weeks. (**A**) 6 min distance. (**B**) Timed up and go. (**C**) Gait speed. Data are presented as Mean ± SD. * *p* < 0.01, ** *p* < 0.001, *** *p* < 0.0001 (significant difference from baseline value). **#** (significant difference between treatment groups).

Figure 3 illustrates that both groups significantly improved in the 6 MWT (F(4,112) = 39.6; *p* < 0.001; ES = 0.59) (Figure 3A) and the TUG (F(4,112) = 22.1; *p* < 0.001; ES = 0.44) (Figure 3B) over 8 weeks compared to the baseline. They gradually began to change after 2 weeks, and the interaction effect of 6 MWT was found (F(4,112) = 4.0; *p* = 0.004; ES = 0.13). There was no group difference in 6 MWT (F(1,28) = 208.4; *p* = 0.38; ES = 0.03) and TUG (F(1,28) = 107.3; *p* = 0.36; ES = 0.03). The gait speed of the 10-meter distance in both groups significantly began to change at 4 weeks (F(1,28) = 50.0; *p* < 0.001; ES = 0.64) (Figure 3C). The gait speed was the single parameter that was significantly higher in the I-Walk than in the conventional PT at 6 and 8 weeks (F(1,28) = 5.9; *p* = 0.022; ES = 0.17) (Figure 3C).

## 4. Discussion

The main finding in this study was that I-Walk training improved gait speed more than conventional PT training. However, I-Walk showed slightly decreased passive joint motion. Improvements of lower extremity impairment and gait performances (gait speed, walking distances, and dynamic balance during walking) were found in both conventional PT training and I-Walk training.

Based on gait speed, the baseline of the community stroke survivors was between 0.4 and 0.8 m/s [5]. The minimally clinically important difference (MCID) was 0.14 m/s in chronic stroke patients [24] and 0.16 m/s in acute stroke patients [25]. The present study showed an increase in gait speed in the range from 0.48 to 0.81 m/s following I-Walk training, which suggested that this training could facilitate the limited community ambulators into unlimited community ambulators [5]. This training also increases gait speed to the upper limit. The experimental group in this study improved in gait speed and FMA-LE score to a greater extent than the group who trained with the I-Walk gait training machine from the previous study [13]. The differences may be due to the severity of the diseases, the patient’s ability, motivation, setting of walking cadence during training, and differences of the gait speed measurement method.

The I-Walk gait training machine sufficiently facilitates locomotor function in chronic stroke in agreement with a previously studied robotic-gait training machine [26,27]. The setting of task-specific locomotor training and increased number of repetitions with normal gait cycle patterns teaches the patient to learn and control new movement with normal biomechanics and to use less energy when performing tasks. Moreover, the number of repetitions with the appropriate gait pattern may be the key point of change in this study. The number of machine and human gait training repetitions in 30 min made a difference for learning control and movement [28]. All the participants in the I-Walk group needed to walk 1500 steps per training session (50 steps/min) with smooth and proper rhythmic movement during gait training. In contrast, the repetitions in the conventional PT group were possibly inadequate to control precise movement. Patient tolerance and ability for constant control movement might be a concern. On the other hand, an important consequence of conventional PT training is sensory stimulation by the therapist. Patient response and range of motion with conventional PT are more effectively enhanced than with machine therapy, as indicated by the higher FMA score. The change of the joint pain sub-score for both groups reached the ceiling effect.

Previous studies found that 1000 steps/training session by robotic device training for 4 weeks over a range of levels of weight bearing on the hindlimbs led to motor recovery in animals with ischemic lesions, [29,30,31] and 291.5 repetitions per session improved the balance and the gait pattern of stroke patients [32]. The repetition of robot-assisted gait training up to 2000 times per session was sufficient to modulate plasticity in the brain and led to the recovery of motor functions, such as postural and locomotion control [28,32]. Even though the repetitions in this study were less than 2000 times per session, they improved locomotor function and balance and reduced lower extremity impairment in chronic stroke. Moreover, 20-session robot-assisted gait training with ankle dorsiflexion assistance induced changes in gait pattern with improved gait independency [33]. From our study, the I-Walk gait training machine allows stroke survivors to practice by themselves in the community. This machine could improve rehabilitation programs and help therapists to easily progress in their training programs. It could also encourage patient confidence in participating in community activities and improve their quality of life.

Gait is a multifactorial task consisting of a healthy cardiovascular system, proper dynamic balance and rhythmic gait speed, and motor control. The 6 MWT is a standard tool widely used for determining walking performance and cardiovascular endurance [34]. Cardiovascular endurance is pivotal in neuroplasticity in stroke survivors [35]. The brain is a vital organ with a high metabolic rate and high oxygen consumption [36]. Efrati et al. found that hyperbaric oxygen therapy induces neuroplasticity in the late stages of stroke [37]. The possible mechanism of oxygen therapy on neuroplasticity is mediated by brain-derived neurotrophic factor (BDNF) [38], insulin-like growth factor-I (IGF-I) [39], and nerve growth factor [40]. Our findings suggest that our improved 6 MWT distances of approximately 126–207 m is consistent with Mustafaoglu et al., who suggested walking distance increases from 25.1 to 78.2 m after gait training for 45 min/day, 2 days/week for 6 weeks [41]. However, Eng et al. observed a greater 6 MWT distance in community-dwelling chronic stroke survivors (267 m) [42]. Therefore, there is still room for improvement, probably by increasing speed through I-Walk repetitive cycles to improve cardiovascular fitness and brain oxygen consumption. Fulk et al. observed the MCID of 6 MWT in chronic stroke patients with an initial gait speed greater than 0.4 m/s with 71 m [43]. Our study suggested an increment of gait speed with the I-Walk training group higher than 1.4 m/s with 81.2 m, which is higher than the MCID [43]. Thus, an improvement in cardiovascular endurance promotes physical performance on dynamic balance during walking training.

Dynamic balance during walking in the community is challenging for individuals with hemiparesis after a stroke [44]. In our study, postintervention dynamic balance development in both groups was measured by the TUG; however, these results did not show a significant difference across groups. Postintervention repetitive task training enhances the learning circuit in the ventral premotor cortex, the inferior parietal lobe, and the insula, resulting in an improvement of lower limb function up to 6 months. We found a higher mean difference for TUG post-training in both groups than the minimal detectable change (MDC; 2.9 s) in stroke patients, [17] indicating clinically significant improvements in dynamic balance [45].

The time spent for TUG in our study significantly decreased in both groups compared to the baseline (15.95 and 21.89 s in the I-Walk and control, respectively), but it is still inadequate to prevent the risk of a fall based on the fall risk cut-off point, i.e., 14 [46] and 15 s [47]. It can be speculated that 30-minute gait training for 8 weeks might correlate with the development of dynamic balance for advanced gait training. Further studies should extend the training time to evaluate gait performance.

Some machine limitations must be noted. First, it did not give complete walking adaptability experiences, which are important for walking in the community [30]. Second, it may be a suitable first approach for training. Third, the ceiling effect of the score has to be considered so that it is close to the upper limit. A strength of the present study is the tendency to improve gait speed and the intensity and repetitions capable of being provided to stimulate posture control and locomotor functions. Moreover, the number of participants in each group provided strength to the results.

## 5. Conclusions

The I-Walk training machine improves gait speed but slightly decreases the range of motion compared to conventional PT training. I-Walk could maximize patient ability for community ambulators; however, it is suitable for first approach training. Consequently, the various environments that need to be altered challenge the patient’s ability. The conventional PT for the gait rehabilitation might have higher potential by including I-Walk training to improve lower limb impairment. The greater intensity and longer duration of this training should be considered for further implementation in the community.

## Figures and Tables

**Table 1:** General characteristics in conventional PT and I-Walk gait training in chronic stroke.

Characteristics	Mean ± SD/Frequency		*p*-Value
	I-Walk (*n* = 15)	Conventional PT (*n* = 15)	
Age (years)	64.33 ± 7.68	63.53 ± 12.16	0.709
Weight (kg)	61.94 ± 9.27	61.73 ± 12.63	0.631
Height (cm)	163.07 ± 8.70	162.33 ± 8.46	0.988
Duration after stroke (years)	6.57 ± 3.57	5.60 ± 5.65	0.580
10 MWT (m/s)	0.48 ± 0.19	0.41 ± 0.17	0.315
6 MWT (m)	126.73 ± 57.61	116.33 ± 55.69	0.619
TUG (s)	27.79 ± 16.31	32.50 ± 18.04	0.460
Total FMA-LE (scores)	85.60 ± 10.15	93.00 ± 11.62	0.074
Sex (Male:Female)	10:5	10:5	
Pathology (Ischemia:Hemorrhage)	11:4	13:2	
Weak side (Right:Left)	7:8	7:8	
Underlying diseases (no disease:1 disease:2 diseases:≥3 diseases)	0:7:3:5	1:2:6:6	

PT: Physical therapy, 10 MWT: 10-meter walk test, 6 MWT: 6-minute walk test, TUG: Timed Up and Go, FMA-LE: Fugl-Meyer assessment of lower extremity.

**Table 2:** Changes of lower extremity impairment scores during 8-week gait training compared within (compared with baseline) and across groups (Conventional PT and I-Walk) in chronic stroke.

Fugl-Meyer Assessment-Lower Extremity (Scores)	I-Walk (*n* = 15)		Conventional PT (*n* = 15)		*p*-Value across Group
	Mean ± SD	*p*-Value	Mean ± SD	*p*-Value	
**Total FMA-LE (86)**					
baseline	71.00 ± 6.72		66.60 ± 6.71		0.083
2-week	72.67 ± 6.32	0.012	70.20 ± 4.55	0.002	0.230
4-week	74.60 ± 5.44	<0.001	72.20 ± 4.96	<0.001	0.217
6-week	77.20 ± 5.89	<0.001	74.53 ± 4.60	<0.001	0.177
8-week	78.67 ± 5.45	<0.001	75.73 ± 4.43	<0.001	0.117
**Lower extremity (28)**					
baseline	18.40 ± 4.14		15.60 ± 2.92		0.042
2-week	19.26 ± 4.06	0.039	17.73 ± 2.05	<0.001	0.202
4-week	20.26 ± 3.57	<0.001	18.60 ± 1.99	<0.001	0.126
6-week	22.00 ± 3.25	<0.001	20.13 ± 2.55	<0.001	0.092
8-week	23.06 ± 3.01	<0.001	20.86 ± 2.66	<0.001	0.043
**Coordination/speed (6)**					
baseline	3.60 ± 1.40		3.40 ± 1.18		0.676
2-week	3.66 ± 1.34	0.543	3.40 ± 1.24	1.000	0.577
4-week	4.13 ± 1.24	0.039	3.93 ± 1.62	0.039	0.708
6-week	4.60 ± 1.24	0.001	4.26 ± 1.33	0.004	0.485
8-week	4.93 ± 1.27	<0.001	4.60 ± 1.18	<0.001	0.465
**Total motor function (34)**					
baseline	22.00 ± 5.07		19.33 ± 3.81		0.115
2-week	22.93 ± 4.93	0.042	21.20 ± 2.78	<0.001	0.246
4-week	24.40 ± 4.35	<0.001	22.53 ± 2.99	<0.001	0.182
6-week	26.60 ± 4.18	<0.001	24.40 ± 3.24	<0.001	0.119
8-week	28.00 ± 4.00	<0.001	25.46 ± 3.33	<0.001	0.070
**Sensation (12)**					
baseline	9.93 ± 2.31		9.40 ± 3.11		0.598
2-week	10.60 ± 2.41	0.053	10.20 ± 2.59	0.022	0.666
4-week	10.86 ± 2.19	0.014	10.40 ± 2.50	0.009	0.592
6-week	11.00 ± 2.23	0.006	10.80 ± 1.82	0.001	0.790
8-week	11.00 ± 2.23	0.008	10.86 ± 1.72	0.001	0.856
**Passive joint motion (20)**					
baseline	19.33 ± 0.81		18.06 ± 0.96		0.001
2-week	19.26 ± 0.88	0.630	18.80 ± 0.86	<0.001	0.154
4-week	19.40 ± 0.73	0.749	19.33 ± 0.72	<0.001	0.804
6-week	19.66 ± 0.40	0.140	19.33 ± 0.72	<0.001	0.150
8-week	19.73 ± 0.45	0.098	19.40 ± 0.73	<0.001	0.148
**Joint pain (20)**					
baseline	19.73 ± 0.59		19.80 ± 0.56		0.754
2-week	19.86 ± 0.35	0.346	20.00 ± 0.00	0.162	0.153
4-week	19.93 ± 0.25	0.162	19.93 ± 0.25	0.346	1.000
6-week	19.93 ± 0.25	0.178	20.00 ± 0.00	0.178	0.326
8-week	19.93 ± 0.25	0.178	20.00 ± 0.00	0.178	0.326

FMA-LE, Fugl-Meyer Assessment-lower extremity; PT = Physical Therapy. Data are presented as Mean ± SD. *p*-values from *t*-test.

## Data Availability

The datasets used and analyzed during this study are available from the corresponding author on reasonable request.

## References

[1] Sharrief A., Grotta J.C. (2019). Stroke in the elderly. Handb. Clin. Neurol..

[2] Mayo N.E., Wood-Dauphinee S., Côté R., Durcan L., Carlton J. (2002). Activity, participation, and quality of life 6 months poststroke. Arch. Phys. Med. Rehabil..

[3] Jette D.U., Latham N.K., Smout R.J., Gassaway J., Slavin M.D., Horn S.D. (2005). Physical therapy interventions for patients with stroke in inpatient rehabilitation facilities. Phys. Ther..

[4] Jørgensen H.S., Nakayama H., Raaschou H.O., Olsen T.S. (1995). Recovery of walking function in stroke patients: The Copenhagen Stroke Study. Arch. Phys. Med. Rehabil..

[5] Perry J., Garrett M., Gronley J.K., Mulroy S.J. (1995). Classification of walking handicap in the stroke population. Stroke.

[6] Ng S.S., Tsang W.W., Cheung T.H., Chung J.S., To F.P., Yu P.C. (2011). Walkway length, but not turning direction, determines the six-minute walk test distance in individuals with stroke. Arch. Phys. Med. Rehabil..

[7] Mayo N.E., Wood-Dauphinee S., Ahmed S., Gordon C., Higgins J., McEwen S., Salbach N. (1999). Disablement following stroke. Disabil. Rehabil..

[8] States R.A., Pappas E., Salem Y. (2009). Overground physical therapy gait training for chronic stroke patients with mobility deficits. Cochrane Database Syst. Rev..

[9] Bizovičar N., Matjačić Z., Stanonik I., Goljar N. (2017). Overground gait training using a motorized assistive device in patients with severe disabilities after stroke. Int. J. Rehabil. Res..

[10] Gama G.L., Celestino M.L., Barela J.A., Forrester L., Whitall J., Barela A.M. (2017). Effects of Gait Training With Body Weight Support on a Treadmill Versus Overground in Individuals With Stroke. Arch. Phys. Med. Rehabil..

[11] Park K.H., Kim D.Y., Kim T.H. (2015). The effect of step climbing exercise on balance and step length in chronic stroke patients. J. Phys. Ther. Sci..

[12] Donath L., Faude O., Roth R., Zahner L. (2014). Effects of stair-climbing on balance, gait, strength, resting heart rate, and submaximal endurance in healthy seniors. Scand. J. Med. Sci. Sports.

[13] Pansuksawat N., Tantilipikorn P., Kooncumchoo P., Rungroungdouyboon B. (2020). Effects of I-Walk training on gait performances in patients with chronic stroke. Vajira Med. J..

[14] Maier M., Ballester B.R., Verschure P. (2019). Principles of Neurorehabilitation After Stroke Based on Motor Learning and Brain Plasticity Mechanisms. Front. Syst. Neurosci..

[15] Kwakkel G., Kollen B.J., Krebs H.I. (2008). Effects of robot-assisted therapy on upper limb recovery after stroke: A systematic review. Neurorehabil. Neural. Repair.

[16] Harada N.D., Chiu V., Stewart A.L. (1999). Mobility-related function in older adults: Assessment with a 6-minute walk test. Arch. Phys. Med. Rehabil..

[17] Flansbjer U.B., Holmbäck A.M., Downham D., Patten C., Lexell J. (2005). Reliability of gait performance tests in men and women with hemiparesis after stroke. J. Rehabil. Med..

[18] Podsiadlo D., Richardson S. (1991). The timed “Up & Go”: A test of basic functional mobility for frail elderly persons. J. Am. Geriatr. Soc..

[19] Gladstone D.J., Danells C.J., Black S.E. (2002). The fugl-meyer assessment of motor recovery after stroke: A critical review of its measurement properties. Neurorehabil. Neural. Repair.

[20] Crow J.L., Harmeling-van der Wel B.C. (2008). Hierarchical properties of the motor function sections of the Fugl-Meyer assessment scale for people after stroke: A retrospective study. Phys. Ther..

[21] Hsueh I.P., Hsu M.J., Sheu C.F., Lee S., Hsieh C.L., Lin J.H. (2008). Psychometric comparisons of 2 versions of the Fugl-Meyer Motor Scale and 2 versions of the Stroke Rehabilitation Assessment of Movement. Neurorehabil. Neural. Repair.

[22] Fugl-Meyer A.R., Jääskö L., Leyman I., Olsson S., Steglind S. (1975). The post-stroke hemiplegic patient. 1. a method for evaluation of physical performance. Scand. J. Rehabil. Med..

[23] Byun S.D., Jung T.D., Kim C.H., Lee Y.S. (2011). Effects of the sliding rehabilitation machine on balance and gait in chronic stroke patients—A controlled clinical trial. Clin. Rehabil..

[24] Perera S., Mody S.H., Woodman R.C., Studenski S.A. (2006). Meaningful change and responsiveness in common physical performance measures in older adults. J. Am. Geriatr. Soc..

[25] Tilson J.K., Sullivan K.J., Cen S.Y., Rose D.K., Koradia C.H., Azen S.P., Duncan P.W. (2010). Meaningful gait speed improvement during the first 60 days poststroke: Minimal clinically important difference. Phys. Ther..

[26] Ada L., Dean C.M., Hall J.M., Bampton J., Crompton S. (2003). A treadmill and overground walking program improves walking in persons residing in the community after stroke: A placebo-controlled, randomized trial. Arch. Phys. Med. Rehabil..

[27] Hidler J.M., Wall A.E. (2005). Alterations in muscle activation patterns during robotic-assisted walking. Clin. Biomech..

[28] Kim S.Y., Yang L., Park I.J., Kim E.J., JoshuaPark M.S., You S.H., Kim Y.H., Ko H.Y., Shin Y.I. (2015). Effects of Innovative WALKBOT Robotic-Assisted Locomotor Training on Balance and Gait Recovery in Hemiparetic Stroke: A Prospective, Randomized, Experimenter Blinded Case Control Study With a Four-Week Follow-Up. IEEE Trans. Neural. Syst. Rehabil. Eng..

[29] Kleim J.A., Barbay S., Nudo R.J. (1998). Functional reorganization of the rat motor cortex following motor skill learning. J. Neurophysiol..

[30] Nudo R.J., Wise B.M., SiFuentes F., Milliken G.W. (1996). Neural substrates for the effects of rehabilitative training on motor recovery after ischemic infarct. Science.

[31] Cha J., Heng C., Reinkensmeyer D.J., Roy R.R., Edgerton V.R., De Leon R.D. (2007). Locomotor ability in spinal rats is dependent on the amount of activity imposed on the hindlimbs during treadmill training. J. Neurotrauma.

[32] Lang C.E., MacDonald J.R., Gnip C. (2007). Counting repetitions: An observational study of outpatient therapy for people with hemiparesis post-stroke. J. Neurol. Phys. Ther..

[33] Yeung L.F., Ockenfeld C., Pang M.K., Wai H.W., Soo O.Y., Li S.W., Tong K.Y. (2018). Randomized controlled trial of robot-assisted gait training with dorsiflexion assistance on chronic stroke patients wearing ankle-foot-orthosis. J. Neuroeng. Rehabil..

[34] Giannitsi S., Bougiakli M., Bechlioulis A., Kotsia A., Michalis L.K., Naka K.K. (2019). 6-minute walking test: A useful tool in the management of heart failure patients. Ther. Adv. Cardiovasc. Dis..

[35] Ploughman M., Austin M.W., Glynn L., Corbett D. (2015). The effects of poststroke aerobic exercise on neuroplasticity: A systematic review of animal and clinical studies. Transl. Stroke Res..

[36] Huchzermeyer C., Berndt N., Holzhütter H.G., Kann O. (2013). Oxygen consumption rates during three different neuronal activity states in the hippocampal CA3 network. J. Cereb. Blood Flow Metab..

[37] Efrati S., Fishlev G., Bechor Y., Volkov O., Bergan J., Kliakhandler K., Kamiager I., Gal N., Friedman M., Ben-Jacob E. (2013). Hyperbaric oxygen induces late neuroplasticity in post stroke patients--randomized, prospective trial. PLoS ONE.

[38] Coelho F.G., Gobbi S., Andreatto C.A., Corazza D.I., Pedroso R.V., Santos-Galduróz R.F. (2013). Physical exercise modulates peripheral levels of brain-derived neurotrophic factor (BDNF): A systematic review of experimental studies in the elderly. Arch. Gerontol. Geriatr..

[39] Gatti R., De Palo E.F., Antonelli G., Spinella P. (2012). IGF-I/IGFBP system: Metabolism outline and physical exercise. J. Endocrinol. Investig..

[40] Chung J.Y., Kim M.W., Bang M.S., Kim M. (2010). The effect of exercise on trkA in the contralateral hemisphere of the ischemic rat brain. Brain Res..

[41] Mustafaoglu R., Erhan B., Yeldan I., Gunduz B., Tarakci E. (2020). Does robot-assisted gait training improve mobility, activities of daily living and quality of life in stroke? A single-blinded, randomized controlled trial. Acta Neurol. Belg..

[42] Eng J.J., Chu K.S., Dawson A.S., Kim C.M., Hepburn K.E. (2002). Functional walk tests in individuals with stroke: Relation to perceived exertion and myocardial exertion. Stroke.

[43] Fulk G.D., He Y. (2018). Minimal Clinically Important Difference of the 6-Minute Walk Test in People With Stroke. J. Neurol. Phys. Ther..

[44] Vistamehr A., Balasubramanian C.K., Clark D.J., Neptune R.R., Fox E.J. (2018). Dynamic balance during walking adaptability tasks in individuals post-stroke. J. Biomech..

[45] Villiger M., Estévez N., Hepp-Reymond M.C., Kiper D., Kollias S.S., Eng K., Hotz-Boendermaker S. (2013). Enhanced activation of motor execution networks using action observation combined with imagination of lower limb movements. PLoS ONE.

[46] Andersson A.G., Kamwendo K., Seiger A., Appelros P. (2006). How to identify potential fallers in a stroke unit: Validity indexes of 4 test methods. J. Rehabil. Med..

[47] Whitney J.C., Lord S.R., Close J.C. (2005). Streamlining assessment and intervention in a falls clinic using the Timed Up and Go Test and Physiological Profile Assessments. Age Ageing.

